# Effects of resveratrol, exercises and their combination on *Farnesoid X receptor, Liver X receptor and Sirtuin 1* gene expression and apoptosis in the liver of elderly rats with nonalcoholic fatty liver

**DOI:** 10.7717/peerj.5522

**Published:** 2018-09-10

**Authors:** Amir Hajighasem, Parvin Farzanegi, Zohreh Mazaheri, Marjan Naghizadeh, Ghoncheh Salehi

**Affiliations:** 1 Department of Exercise Physiology, Sari Branch, Islamic Azad University, Sari, Iran; 2 Department of Anatomical Sciences, Faculty of Medical Sciences, Tarbiat Modares University, Tehran, Iran

**Keywords:** Resveratrol, Interval exercise, NAFLD, Continuous exercise, *Sirt1*, *Lxr*, *Fxr*

## Abstract

**Background:**

Non-alcoholic fatty liver disease (NAFLD) is the most common chronic liver disorder. This study aims to consider effects of resveratrol, exercise and their combination on *Farnesoid X receptor (*Fxr*), the liver X receptor (*Lxr*) and Sirtuin 1 (Sirt 1)* genes expression in the liver of elderly rats with NAFLD.

**Methods:**

Rats with NAFLD were randomly divided into seven groups including patient, saline, resveratrol (RSV), interval exercise, continuous exercise, interval exercise + RSV and continuous exercise + RSV. Levels of alanine aminotransferase (ALT), aspartate aminotransferase (AST) and alkaline phosphatase (ALP) in the liver tissue were measured using specific ELISA kits. A TUNEL assay kit was used for the assessment of hepatic cells apoptosis. Lipid profiles were considered by measuring the serum triglyceride, cholesterol, LDL, and HDL. Expression of *Sirt1*, *Lxr* and *Fxr* genes was considered using RT-PCR.

**Results:**

Resveratrol administration alone or combined with exercise training significantly improved the expression of *Sirt1*, *Lxr* and *Fxr* genes (*p* < 0.05) in the hepatic tissue of rats with NAFLD, while levels of AST, ALT, ALP enzymes, as well as apoptotic cells were significantly decreased (*p* < 0.05).

**Discussion:**

Although resveratrol alone improves the expression of *Sirt1*, *Lxr* and *Fxr*, as well as liver function, combined therapy with exercise training is more effective to improve NAFLD.

## Introduction

Non-alcoholic fatty liver disease (NAFLD) is a common type of chronic hepatic disease that affects 25–30% of people in the world (Li et al., 2015). It is associated with a significant accumulation of lipids in the liver and consequently severe abnormalities in liver enzymes (Abd El-Kader & El-Den Ashmawy, 2015). NAFLD not only causes severe damage to the liver, but also it is associated with secondary complications such as overweight, dyslipidaemia, diabetes mellitus, coronary and cardiovascular diseases (Lomonaco et al., 2013). Therefore, a large number of evidences have considered the molecular mechanisms of NAFLD pathogenesis and its therapeutic strategies (Abd El-Kader & El-Den Ashmawy, 2015).

Numerous factors such as gender, fatness, diabetes, age, and ethnicity have been shown to be responsible for NAFLD development (Clark, Brancati & Diehl, 2003; Schwimmer, 2007). Recent studies have indicated that NAFLD can be correlated to expression of various genes such as Farnesoid X receptor (*Fxr*), the liver X receptor (*Lxr*) and Sirtuin 1 (*Sirt1*). *Sirt1* is an NAD^+^-dependent deacetylase which acts as a regulator for different metabolic pathways such as glucose homeostasis, lipid mobilization, *β*-oxidation, oxidative stress, insulin secretion and sensitivity, inflammation, cellular aging and apoptosis (Colak et al., 2011). Recent studies have shown that *Sirt1* has a hepatoprotective effect against NAFLD; however, its expression in the liver is significantly decreased in NAFLD model of rats fed with high-fat diet (Colak et al., 2011). *Fxr* is a ligand-activated transcription factor that is abundantly expressed in the liver. The hepatoprotective role of *Fxr* is essential for normal liver function as it plays a critical role in regulating lipid metabolism and suppressing inflammation in the liver (Zhu et al., 2016). *Lxr* is an oxysterol-activated nuclear receptor which is responsible for the regulation of major metabolic pathways for cholesterol homeostasis, bile acid metabolism and lipogenesis (Ahn et al., 2014). A great number of studies have reported decreased expression of these genes in the liver of patients with NAFLD. Therefore, use of drugs or natural compounds to induce *Sirt1, Fxr and Lxr* expression may provide new opportunities to improve the pathogenesis of NAFLD.

Resveratrol (RSV) is a type of natural phenol compound produced by different plants, such as berries and grape skins (Baur & Sinclair, 2006). Numerous studies showed that resveratrol is able to mitigate oxidative stress by improving antioxidant defense system, preventing synthesis and release of pro-inflammatory cytokines, modulating synthesis of eicosanoids, and preventing nicotinamide adenine dinucleotide phosphate (NADPH) oxidases and activity of cyclooxygenase-2 (COX-2) (Messina et al., 2015).

Recent evidence has also revealed that regular physical activities can protect body organs against a wide range of acute and chronic diseases (Chicco et al., 2006). Regular physical training has also protective effects against various abnormalities such as aging, oxidative stress, inflammation and apoptosis (Chicco et al., 2006). More recently, we have found that combined therapy with resveratrol and interval or continuous exercises decreases oxidative stress, inflammation and hepatic cells apoptosis in the livers of rats with NAFLD, while this combined therapy improves antioxidant defense and anti-inflammatory systems (Hajighasem, Farzanegi & Mazaheri, 2018). However, the exact mechanism in which this combined therapy improves hepatic cell apoptosis, antioxidant and anti-inflammatory systems is unclear. Since *Sirt1, Fxr* and *Lxr* regulate lipid metabolism, as well as oxidative stress and inflammation in the liver, the expression of these genes is very important in NAFLD pathogenesis. We assume that overexpression of *Sirt1, Fxr and Lxr* genes may be a main possible mechanism by which combined therapy with resveratrol and exercises improve NAFLD. Recent evidences have shown correlation between lipid profiles, oxidative stress, inflammation, apoptosis and expression pattern of *Lxr*, *Fxr* and *Sirt1* genes in the liver. Since we found that RSV alone or in combination with exercise positively affects these parameters, in the present work we hypothesized that increased levels of oxidative stress, inflammation and apoptosis, which were seen in NAFLD rats in our previous study (Hajighasem, Farzanegi & Mazaheri, 2018), may be due to decreased expression of *Lxr*, *Fxr* and *Sirt1*. Therefore, we decided to consider this correlation in NAFLD rats and also the effects of treatments with RSV and exercises on these correlations. Since we have had a fraction of liver tissue from the same experimental groups stored at −80, we have performed the present work with the same experimental groups. Therefore, in line with our recent study, we aim to consider the effect of resveratrol supplementation along with interval and continuous exercises on *Sirt1*, *Fxr* and *Lxr* genes expression, lipid profiles, liver injuries, and apoptosis in the liver of rats with moderate NAFLD.

## Materials and Methods

### Animals and NAFLD induction

56 male elderly Wistar rats (mean age of 40–50 weeks and a body weight of 250–300 g) were randomly isolated from laboratory of animal research center at the Islamic Azad University of Sari-Iran. All rats were housed 4 per cage (30 × 15 × 15 cm) in an air controlled room (temperature of 22 ± 2 °C, humidity of 50 ± 5%, and a 12:12 light/dark cycle). This research was approved by the animal care and use committee at the Islamic Azad University of Sari (Approval reference number: IR.IAU.SARI.REC.1395.54). Rats in NAFLD group were fed with a high fat diet (HFD) (22% fat, 2% cholesterol, 1% choline, 50% carbohydrate, 24% protein and 1% other compounds) for 6 weeks to induce moderate NAFLD according to our recent study (Hajighasem, Farzanegi & Mazaheri, 2018), while rats in the control group received a standard diet (12% fat, 57% carbohydrate, 28% protein and 3% other compounds) (Efati et al., 2016). The histopathological examinations and other biochemical findings such as biomarkers of oxidative stress and inflammatory in NAFLD rats can be seen in our recent published work (Hajighasem, Farzanegi & Mazaheri, 2018). The HFD rats were randomly divided into seven groups (7 rats in each group) including: patient (only fed with HFD), saline, resveratrol, continuous exercise, interval exercise, continuous exercise + resveratrol, and interval exercise + resveratrol groups. Rats in the resveratrol group received 25 mg/kg resveratrol daily through intraperitoneal injection. Rats in control, patient and saline groups didn’t receive resveratrol or exercise training.

### Training program

To minimize stress, rats in exercise training groups were familiarized with a rodent treadmill for five consecutive days (with a speed of 10 m/min at 0% inclination for 5 min/day) (Batacan Jr et al., 2016; Freitas et al., 2017). The exercise training program is described in our recent study (Hajighasem, Farzanegi & Mazaheri, 2018). Briefly, during the beginning and end of interval and continuous exercises training, a warm-up and cool-down time was provided at 5 m/min. Rats in interval training group exercised 3 days/week for eight weeks. However, the speed and time of exercises were gradually increased by 2 m/min and 2 minutes, respectively per week. Finally, the speed of training reached 28 m/min in the last week. Rest times were provided as two minutes between each interval. Rats in continuous training group exercised 5 days/week for 8 weeks. Rats were exercised for 5 minutes at a velocity of 15 m/min in the initial week. The speed and time of training were gradually increased by 1–2 m/min and 1–2 minutes, respectively per week. Eventually, the speed of exercise training reached 28 m/min in the last week.

### Liver enzymes assay

Rats were anesthetized with ketamine (30–50 mg/kg) and xylasine (3–5 mg/kg) 48 hours after the last training. The liver tissue of all rats was separated and stored at −80 °C until further consideration. Blood samples were directly collected from the abdominal aorta for the assessment of liver enzymes, including alanine aminotransferase (ALT), aspartate aminotransferase (AST) and alkaline phosphatase (ALP). Activity of these enzymes was measured using commercial enzymatic assay kits (Cod: 119600R910, 118600R910, and 102600R910, respectively) based on instructions provided from Pars Azmun Company (Tehran, Iran).

### Lipid profiles

Content of serum lipids, including HDL, LDL, cholesterol (Cho), and triglyceride (TG) in all rats were measured using specific kits purchased from Pars Azmun Company.

### Detection of apoptosis by TUNEL assay

Detection of hepatic apoptosis in all samples was provided using a TUNEL (Terminal deoxynucleotidyl transferase dUTP nick end labeling) assay Kit (In Situ Cell Death Detection Kit, POD; Roche, Germany).

### RNA isolation and cDNA synthesis

Liver samples were homogenized in phosphate buffer (pH 7.0) at 4 °C with homogenizer (Ma et al., 2017). Total RNAs were extracted from liver tissues of all rats using the RNX-Plus (SinaClon; RN7713C) Kit. Nanodrop ND-1000 spectrophotometer (Thermo Sci., Newington, NH) method was applied to estimate the quantity and quality of extracted RNAs.

### Real-time PCR

RNA samples were transcribed using Revert Aid Reverse Transcriptase (Thermo science, Germany) at 42 °C for 1 h and random hexamer primers (Thermo science, Germany). A Rotor Gene 6000 (Corbett Research, Australia) thermocycler and Real Q-PCR 29 Master Mix Kit (Amplicon, Denmark) in 40 cycles were applied for amplifications. Each reaction included 5 µl master mix and 100 nm primers. Primer sequences were synthesized as follows: *Fxr*, 5′-AGTTGGAAAGTTGGAGTG-3′(forward), 5′-GATTGTTGTATGGGGAGTA-3′(reverse); *Lxr*, 5′-CTGATTCTCCGTGTCCTCTGTG-3′(forward), 5′-CACCCTACCCTTTGACTCTCT-3′(reverse), and *Sirt1*, 5′-GAGTTGTGT GTAGGTTAGGTGG-3′(forward); 5′-AAATATGAAGAGGTGTTGGTGG-3′(reverse); glyceraldehyde 3-phosphate dehydrogenase (GAPDH), 5′-AAGTTCAACGGCACAGTCAA GG-3′(forward); 5′-CATACTCAGCACCAGCATCACC-3′(reverse). The levels of mRNA were normalized relative to the amount of GAPDH mRNA.

### Statistical analysis

Data are presented as means ± SD. The mean of all parametric data between the groups was compared using the One-Way ANOVA: Post Hoc-Tukey test. Data were analyzed using SPSS software (version 19). A *p* < 0.05 was considered significant.

## Results

### Liver enzymes

**Figure 1:**
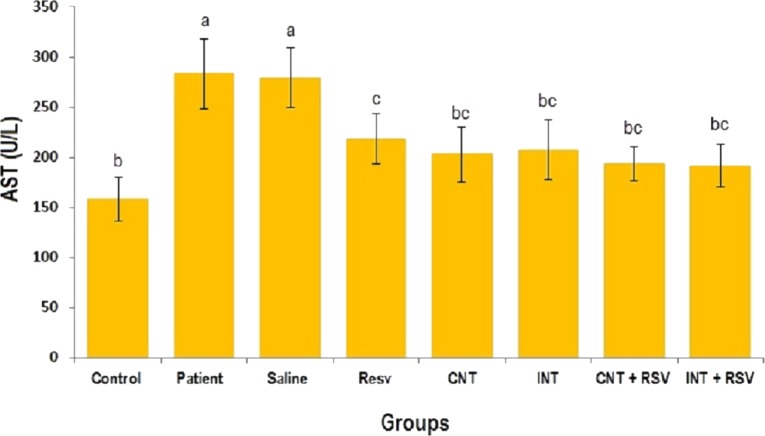
Mean of AST value in each group. There was no significant difference in mean level of AST between groups with similar symbols ((a), (bc and b), (bc and c)). The mean concentration of AST was in order a > c > bc > b. One-Way ANOVA: Post Hoc-Tukey test was applied to compare mean value of parameters between all groups. *p* < 0.05 is considered significant; Resv, resveratrol; CNT, continuous exercise; INT, interval exercise; CNT + RSV, continuous exercise + resveratrol; INT + RSV, interval exercise + resveratrol.

**Figure 2:**
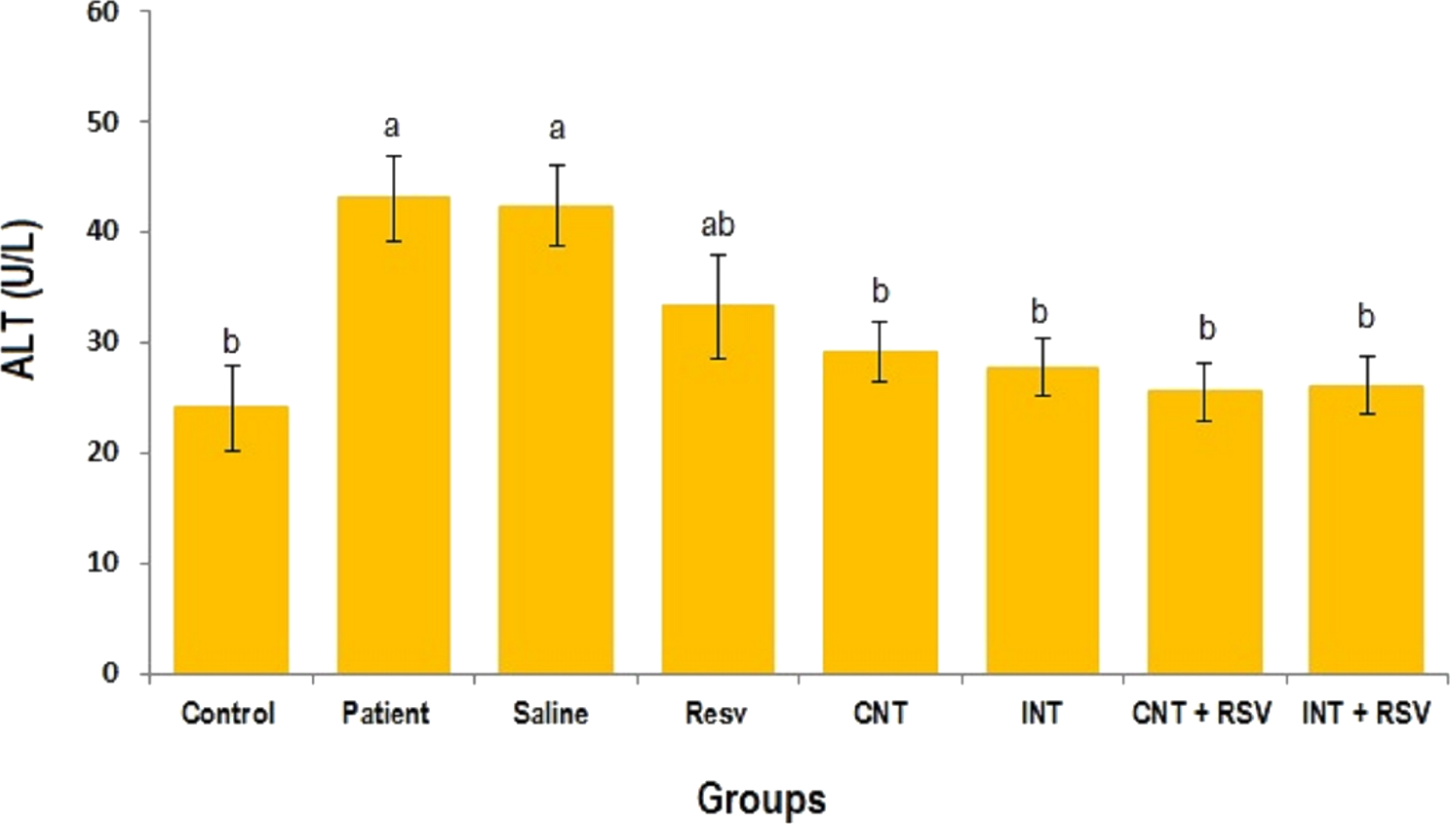
Mean of ALT value in each group. There was no significant difference in mean level of ALT between groups with similar symbols ((a), (a and ab), (b) (ab and b)). The mean concentration of ALT was in order a > ab > b. One-Way ANOVA: Post Hoc-Tukey test was applied to compare mean value of parameters between all groups. *p* < 0.05 is considered significant; Resv, resveratrol; CNT, continuous exercise; INT, interval exercise; CNT + RSV, continuous exercise + resveratrol; INT + RSV, interval exercise + resveratrol.

**Figure 3:**
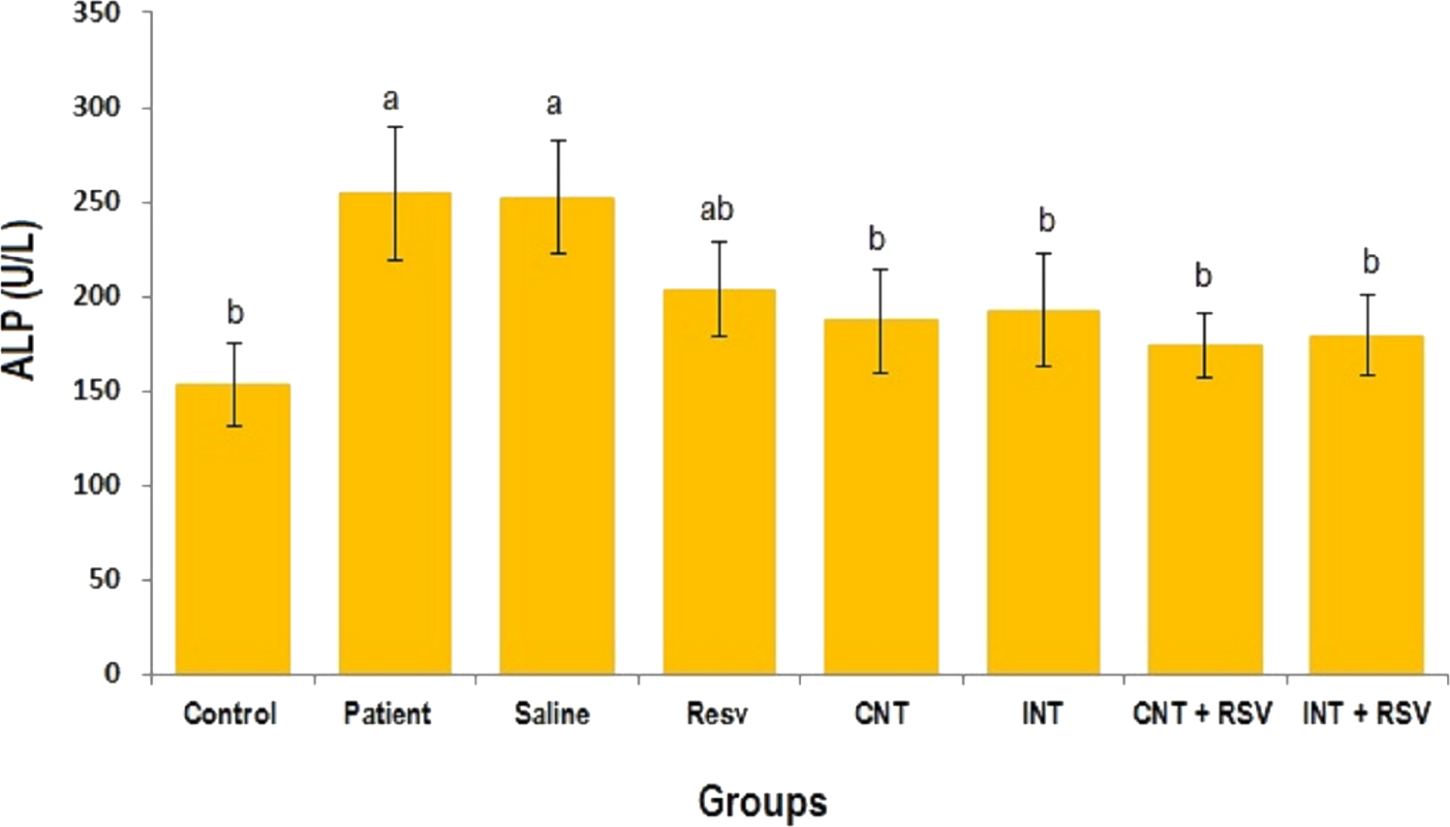
Comparison of the mean of ALP value between each group. There was no significant difference in mean level of ALP between groups with similar symbols ((a), (a and ab), (b) (ab and b)). The mean concentration of ALP was in order a > ab > b. One-Way ANOVA: Post Hoc-Tukey test was applied to compare mean value of parameters between all groups. Resv: resveratrol; CNT, continuous exercise; INT, interval exercise; CNT + RSV, continuous exercise + resveratrol; INT + RSV, interval exercise + resveratrol. Results are presented as Mean ± SE; *p* < 0.05 is considered significant.

The mean levels of AST, ALT and ALP enzymes in serum of all groups are presented in Figs. 1–3. A significant difference was observed in mean levels of AST, ALT and ALP between the groups (*p* = 0.001). The mean values of AST (Fig. 1), ALT (Fig. 2) and ALP (Fig. 3) in patient (283.4 ± 44.9, 43.1 ± 2.9, and 254.4 ± 34.9 U/L, respectively) and saline (283.4 ± 43.8, 42.5 ± 2.7, and 252.3 ± 29.9 U/L, respectively) groups were significantly higher than in the control (*p* < 0.0001) group. Combined therapy with resveratrol supplementation and interval or continuous training significantly reduced the activity of these enzymes compared to the control and saline groups (*p* < 0.001). Although resveratrol or exercise trainings alone could decline the activity of these hepatic enzymes, combined therapy with resveratrol supplementation and exercise training was more effective.

### Lipid profiles

The mean of lipid contents in all groups are summarized in Table 1. A significant difference was found in means of HDL, LDL, Ch and TG levels between all groups (*p* < 0.0000). Patient and saline groups showed higher LDL, Cho and TG mean values compared to the other groups (*p* < 0.0001). The mean of serum HDL in patient (23.24 ± 5.41 mg/dl) and saline (22.98 ± 5.5 mg/dl) groups was significantly lower than that in other groups (*p* < 0.0001). Combined therapy with resveratrol and exercise training significantly decreased the mean of serum LDL, Cho and TG contents compared to the patient and saline groups (*p* < 0.0001). Furthermore, resveratrol alone or in combination with interval or continuous exercises increased the mean value of HDL compared to saline and patient groups (*p* < 0.0001). Non-significant difference was found in mean of LDL, Cho and TG between resveratrol, interval and continuous exercise groups. Additionally, there was no significant difference in mean of serum HDL contents between resveratrol, interval exercise, resveratrol + exercise training groups (Table 1).

**Table 1:** Lipid profile pattern in different groups.

**Groups**	**HDL (mg/dl)**	**LDL (mg/dl)**	**Cho (mg/dl)**	**TG (mg/dl)**
Control	36.54 ± 5.76^a^	23.64 ± 5.27^c^	81.91 ± 10.25^c^	104.64 ± 17.6^d^
Patient	23.24 ± 5.41^b^	48.9 ± 8.94^a^	124.67 ± 10.8^a^	228.18 ± 20.02^a^
Saline	22.98 ± 5.5^b^	50.7 ± 9.32^a^	125.38 ± 13.23^a^	227.42 ± 18.94^a^
Resveratrol	31.15 ± 4.98^a^	34.24 ± 7.01^b^	98.98 ± 14.03^b^	149.9 ± 25.7^bc^
Continuous exercise	28.52 ± 5.48^ab^	39.58 ± 4.91^b^	96.94 ± 21.11^b^	164.92 ± 20.87^b^
Interval exercise	30.97 ± 6.04^a^	37.54 ± 6.21^b^	94.18 ± 15.29^bc^	156.07 ± 19.24^b^
Continuous exercise + RSV	33.32 ± 5.58^a^	31.31 ± 7.57^c^	90.34 ± 12.55^bc^	140.61 ± 15.09^c^
Interval exercise + RSV	34.57 ± 7.58^a^	29.57 ± 6.93^c^	87.94 ± 13.18^bc^	133.92 ± 13.02^c^
*p*-value	<0.0000	<0.0000	<0.0000	<0.0000

**Notes.**

There was no significant difference in mean of lipid profiles between groups with similar symbols. The means of lipid profiles were in order a>ab>b>bc>c. One-Way ANOVA: Post Hoc-Tukey test was applied to compare mean value of parameters between all groups. *p* < 0.05 is considered significant; RSV, Resveratrol. HDL, High density lipoproteins; LDL, Low density lipoproteins; Ch, Cholesterol; TG. Triglycerides; ^∗^*p* < 0.05 is considered significant.

**Table 2:** The percentage of apoptotic cells in each group.

**Groups**	**Apoptotic cells (%)**
Control	10.1 ± 0.98^a^
Patient	34.68 ± 1.44^f^
Saline	32.7 ± 1.5^f^
Resveratrol	17.12 ± 0.48^c^
Continuous exercise	24.54 ± 0.39^e^
Interval exercise	21.3 ± 0.6^d^
Continuous exercise + RSV	14.85 ± 0.32^b^
Interval exercise + RSV	10.74 ± 0.83^a^
*p*-**value**	*P* < 0.0001

**Notes.**

There was no significant difference in mean percentage of apoptotic cells between groups with similar symbols (a–f). The percentage of apoptotic cells were in order f>e>d>c>b>a. One-Way ANOVA: Post Hoc-Tukey test was applied to compare mean value of parameters between all groups. *p* < 0.05 is considered significant; RSV, Resveratrol.

### TUNEL assay

There was a significant difference in mean levels of apoptotic cells between all groups (Table 2; *p* < 0.001). The patient (31.44%) and saline (31.29%) groups had significantly higher percentages of apoptotic hepatic cells than other groups (*p* < 0.001). Resveratrol administration significantly decreased the percentage of apoptotic cells (17.12%); however, its combination with interval (10.74%) and continuous (14.85%) exercises was more effective to decrease the percentage of apoptotic cells.

### Expression of *LXR*, *FXR* and *SIRT1* genes

**Figure 4:**
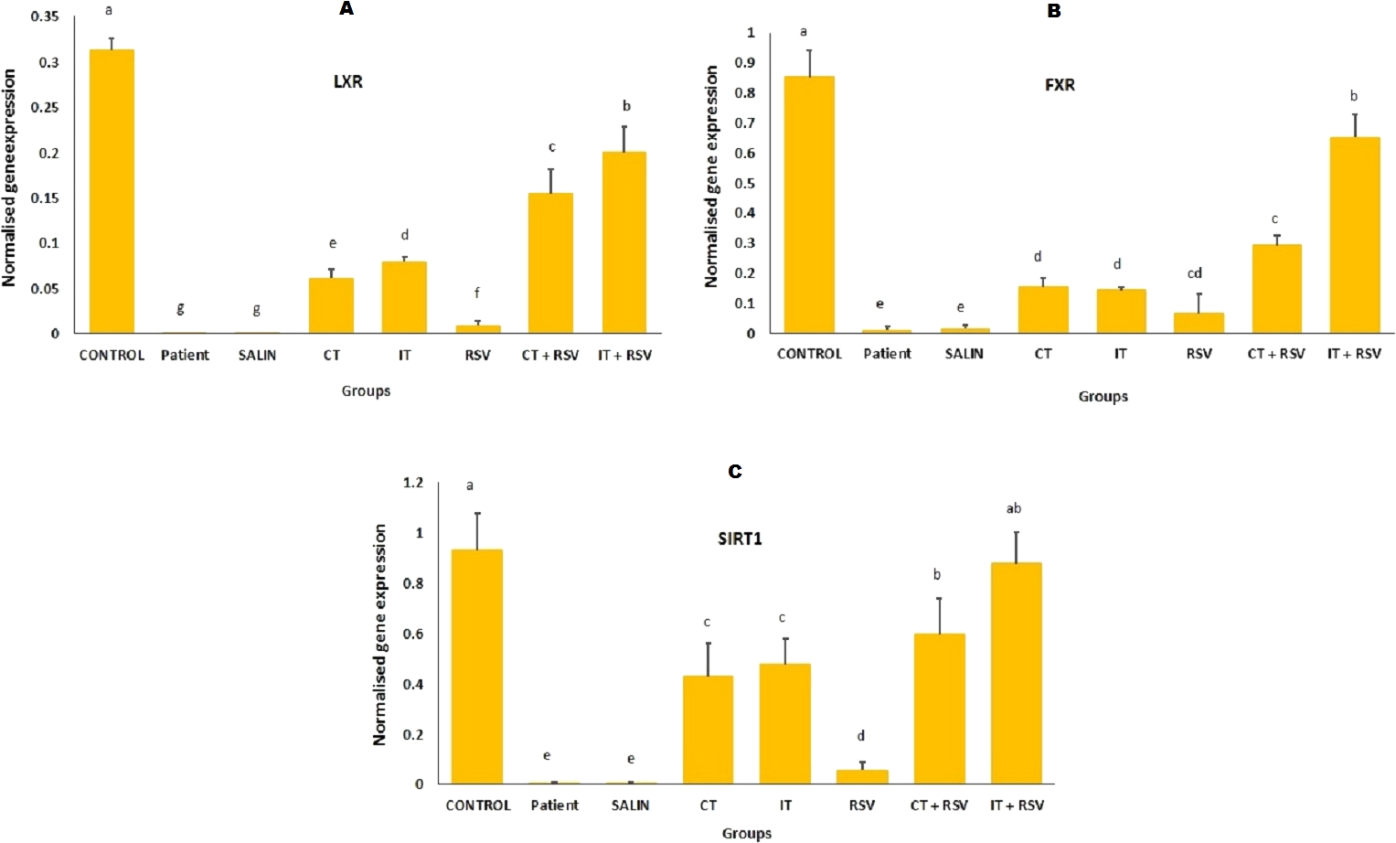
Comparison of the mean mRNA levels of *LXR*, *FXR*, and *SIRT1* were detected by RT-PCR. There was no significant difference in the mRNA levels of *LXR*, *FXR*, and *SIRT1* between groups with similar symbols ((a), (a and ab), (b), (c), (d) (d and cd), (e), (f), (g)). The mean mRNA levels of *LXR*, *FXR*, and *SIRT1* were in order a > ab > b > c > cd > d > e > f > g. One-Way ANOVA: Post Hoc-Tukey test was applied to compare mean value of parameters between all groups. *p* < 0.05 is considered significant; Resv, resveratrol; CNT, continuous exercise; INT, interval exercise; CNT + RSV, continuous exercise + resveratrol; INT + RSV: interval exercise + resveratrol.

The control group showed significantly elevated mRNA expression of *LXR*, *FXR* and *SIRT1* as compared with the other groups (Fig. 4; *p* < 0.001). The mRNA expression of *LXR* (Fig. 4A)*, FXR* (Fig. 4B), and *SIRT1* (Fig. 4C) in the resveratrol and exercise training groups was higher than those in patient and saline groups (Fig. 4; *P* < 0.01). More importantly, increases in the mRNA levels of *FXR*, *LXR* and *SIRT1* in rats treated with a combination of resveratrol and exercise training were more significant than those treated with resveratrol and exercise alone (Fig. 4; *p* < 0.001).

## Discussion

In this research, we evaluated the positive effect of resveratrol supplementation, exercise training and their combination on *Sirt1*, *Lxr* and *Fxr* gene expression, hepatic specific enzymes, lipid profiles and hepatic cell apoptosis in old rats with moderate NAFLD. Our data shows reduced expression of *Sirt1*, *Lxr* and *Fxr* genes in the liver of elderly rats with NAFLD. A great number of studies have shown that NAFLD is associated with decreased expression and inappropriate function of *Sirt1*, *Lxr* and *Fxr* genes (Deng, Chen & Li, 2007; Fiorucci et al., 2011; Fuchs, 2012). For example, McGettigan et al. (2016) indicated that expression of genes involved in *Fxr* signaling in the liver is significantly changed in mice with NAFLD. Moderate expression of *Sirt1* has been reported to protect mice from developing NAFLD (Colak et al., 2011). In a recent study, mice with ablated *Sirt1* catalytic activity showed significantly higher liver triglycerides (Cheng et al., 2017). Furthermore, the mRNA level of lipogenic genes, hormone sensitive lipase, adipose triglyceride lipase and perilipin-2 significantly increased in mesenteric adipose tissue (Cheng et al., 2017). In another study, Nascimento et al. (2013) indicated that *Sirt1* suppression is responsible for alcohol-exacerbated hepatic inflammation and apoptosis in rats with NAFLD. In other research, Sim et al. (2014) demonstrated that *Lxr* expression is correlated with the degree of hepatic fat deposition, hepatic inflammation and fibrosis in NAFLD patients.

Our data also revealed that reduced expression of *Sirt1*, *Lxr* and *Fxr* is associated with increased levels of serum lipids, ALT, AST and ALP in rats with NAFLD. Several lines of studies indicated NAFLD is closely related to increased levels of LDL, TG, Cho, and AST, ALT and ALP values (Mousavi et al., 2017; Tasneem, Luck & Majid, 2018). For instance, El-Din et al. (2014) found high levels of serum ALT, AST, ALP, leptin, total Cho, and TG in the livers of NAFLD subjects. Interestingly, we found that reduced expression of *Sirt1*, *Lxr* and *Fxr* is associated with a significant increase in the percentage of hepatic apoptotic cells in NAFLD rats. Previous research showed that NAFLD is closely related to the activation of mitochondrial-dependent cell death and apoptosis (Ibrahim, Kohli & Gores, 2011; Kalsch et al., 2011). Therefore, these data suggest that reduced expression of *Sirt1*, *Lxr* and *Fxr* can be considered a main reason in the pathogenesis of NAFLD which can be associated with liver damage and apoptosis. Recent studies have demonstrated that *Sirt1*, *Lxr* and *Fxr* agonists or inducers can improve NAFLD (Sanyal, 2015). It has been suggested that the impact of *Fxr* transcriptional activity in NAFLD is likely to be a potential therapeutic strategy (Kim et al., 2016; Zheng et al., 2017). For example, Ren, Huang & Cheng (2014) demonstrated that blueberry juice and *bifidobacteria* cause NAFLD improvement by activating *Sirt1*-mediating signaling pathways. In another study, Xu et al. (2013) showed that metformin can increase the expression of *Sirt1* in the liver of rats with T2DM and NAFLD. In a more recent study with the same experimental subjects, we have shown that moderate NAFLD is associated with histological changes, increased levels of oxidative stress, inflammatory reactions, and apoptosis in the liver tissue of male old rats with moderate NAFLD (Hajighasem, Farzanegi & Mazaheri, 2018). In the current research, we have found that moderate NAFLD is also associated with reduced expression of *Lxr*, *Fxr*, and *Sirt1* as well as increased levels of hepatic injury biomarkers. These data suggest that NAFLD is a multifactorial disease.

Given the critical roles of these genes in NAFLD pathogenesis, oxidative stress, inflammation and lipid metabolism, they can be a target for different drugs to protect hepatocytes. Here, we compared the effects of resveratrol, interval and continuous exercises and their combination to enhance *Sirt1*, *Lxr* and *Fxr* gene expression in NAFLD rats. In the present study, liver injuries were significantly decreased in NAFLD rats after resveratrol administration. We found that the expression of *Sirt1*, *Lxr* and *Fxr* was significantly increased in rats treated with resveratrol. Furthermore, this effect was associated with a significant decrease in serum LDL, TG, Cho, AST, ALT and ALP contents, and hepatic apoptotic cells. Similarly, previous studies reported that resveratrol supplement can improve NAFLD through overexpression of *Sirt1* and activation of AMPK-alpha pathway (Goh et al., 2014; Wang et al., 2016). A more recent study has considered resveratrol as an *Fxr* agonist that may act as a potential compound for the treatment of drug-induced cholestasis (Ding et al., 2018). In another study, Sevov, Elfineh & Cavelier (2006) showed that resveratrol induces *Lxr*-alpha in human monocyte-derived macrophages. In the current research, we observed that resveratrol supplementation combined with exercise training can be more effective. This combination therapy significantly increased the expression of *Sirt1*, *Lxr* and *Fxr* genes and decreased lipid and apoptotic cell contents compared to rats treated with resveratrol alone. This combined therapy also reduced serum concentrations of AST, ALT and ALP enzymes. Several studies evaluated the positive effect of resveratrol combined with different exercise on liver tissue in NAFLD patients. For example, Faghihzadeh et al. (2014) showed that combining resveratrol supplementation (500 mg/day for 12 weeks) with exercise training is associated with a significant decrease in ALT. Tung et al. (2015) showed that both resveratrol and exercise or their combination increase the activity of antioxidant defense systems and protect cells against free radicals. A more recent study has shown that exercise, resveratrol and their combination have protective effects against Sarcopenia, an age-related syndrome, by increasing the expression of p-AMPK and *Sirt1* (Liao et al., 2017). Therefore, our findings suggest that combined therapy with resveratrol supplementation and exercise training can be more appropriate to improve *Sirt1*, *Lxr* and *Fxr* expression, as well as hepatic cell function, lipid profiles and apoptosis in rats with NAFLD. However, the small number of rats in the subgroups is a limitation of our study.

## Conclusions

The results of the current study revealed that NAFLD is closely associated with reduced expression of *Sirt1*, *Lxr* and *Fxr* genes, abnormal lipid profiles, liver cell injuries and apoptosis. Resveratrol alone or combined with exercise training increased the expression of these genes in the livers of NAFLD rats which was subsequently associated with decreased levels of liver specific enzymes, LDL, TG, Cho and hepatic apoptotic cells. Therefore, pharmacologic activation of *Sirt1*, *Lxr* and *Fxr*, which has been implicated in the pathogenesis of NAFLD, may be a potential therapeutic target for treating NAFLD. Although resveratrol has antioxidative and anti-inflammatory properties, its combination with physical activities can be more effective to decrease NAFLD-induced abnormalities.

##  Supplemental Information

10.7717/peerj.5522/supp-1 Data S1 Raw data for gene expression and oxidative stress

## Additional Information and Declarations

### Data Availability

The following information was supplied regarding data availability: The raw data are provided in Data S1.
